# Comparing the retention mechanisms of tandem duplicates and retrogenes in human and mouse genomes

**DOI:** 10.1186/1297-9686-42-24

**Published:** 2010-06-28

**Authors:** Zhen Wang, Xiao Dong, Guohui Ding, Yixue Li

**Affiliations:** 1 Key Lab of Systems Biology, Shanghai Institutes for Biological Sciences, Chinese Academy of Sciences, 320 Yueyang Road, Shanghai, PR China; 2 Graduate School of the Chinese Academy of Sciences, 19 Yuquan Road, Beijing, PR China; 3 Shanghai Center for Bioinformation Technology, 100 Qinzhou Road, Shanghai, PR China

## Abstract

**Background:**

Multiple models have been proposed to interpret the retention of duplicated genes. In this study, we attempted to compare whether the duplicates arising from tandem duplications and retropositions are retained by the same mechanisms in human and mouse genomes.

**Results:**

Both sequence and expression similarity analyses revealed that tandem duplicates tend to be more conserved, whereas retrogenes tend to be more divergent. The duplicability of tandem duplicates is also higher than that of retrogenes. However, positive selection seems to play significant roles in the retention of both types of duplicates.

**Conclusions:**

We propose that dosage effect is more prevalent in the retention of tandem duplicates, while 'escape from adaptive conflict' (EAC) effect is more prevalent in the retention of retrogenes.

## Background

Gene duplication is one of the most important sources of genomic novelty and complexity [1]. There are three main molecular mechanisms leading to new duplicates [2,3]: 1) unequal crossing-over during homologous recombination, 2) duplicative transposition at the DNA level and retroposition mediated by mRNA, and 3) polyploidization. While polyploidization is characterized by bursts of large-scale genome duplication, the former two processes are often small-scale and proceed continuously [4]. Recently, the investigations of full genome sequences have revealed that both large- and small-scale duplications play significant roles in the evolution of various organisms [5]. Although the molecular basis of gene duplication has been well understood, how the newly created duplicates are fixed in the population is still quite controversial [6]. Several evolutionary models for this issue have been proposed, and according to the current perspective [3], they can be distinguished from two independent dimensions: 1) the extent of functional divergence for the new duplicates, and 2) whether positive (adaptive) selection is involved in the process. The outcomes of functional divergence are usually classified as gene conservation, subfunctionalization and neofunctionalization [2,3], though the definitions for the latter two are often ambiguous. Theoretically, the duplicates can undergo adaptive evolution or neutral genetic drift to achieve each outcome.

Statistical analyses on empirical data have suggested that none of the mechanisms alone can interpret the maintenance of all duplicates [3]. However, we suspect that these retention mechanisms may not contribute equally for duplicates stemming from different molecular bases. In fact, by examining the substitution rate between duplicated pairs, Jun et al. [7] have found that retrotransposed and interspersed segmental duplicates diverge more quickly than tandem duplicates. To further compare the underlying retention mechanisms, we attempted to investigate the tandem duplicates arising from unequal crossing-over and retrogenes arising from retroposition in human and mouse genomes. We chose both types of duplicates because: 1) tandem duplicates and retrogenes are easier to screen, and 2) after ancient large-scale genome duplications at the origin of vertebrates, most duplicates have been created via small-scale events in mammalian genomes [8]. In addition, we made the assumption that the duplication rate for each type is constant per year rather than per generation in mammalian genomes. This seems a reasonable assumption because the duplication rate has often been presented with respect to absolute time scale in previous studies [9,10].

## Methods

### Collection of duplicates

All paralogs (protein-coding genes with pseudogenes excluded) and relevant annotations (identity scores, locations and exons) were retrieved from Ensembl database (release 50) via BioMart [11], which amounted to 80,683 and 159,047 pairs of duplicates in human and mouse genomes, respectively. The original dataset had a lot of redundant pairs in multi-member gene families. For example, in a *n*-member family, there would be *n*(*n*-1)/2 paralogous pairs listed, although at most only *n*-1 duplication events were needed to create the family. In this case, we only chose the *n*-1 pairs that contained all the members and had the highest total identity score. Altogether, 9,425 and 11,224 non-redundant pairs were preserved for the two genomes. Next, we applied CHSMiner [12] to detect and remove paralogous segments arising from large-scale duplications. The segments should contain at least two pairs of duplicates, and the gap size between two neighbouring duplicates in either segment should be less than 30 genes [13]. The duplicated pairs located in those segments with FDR < 0.05 were filtered. After this step, we obtained 6,552 and 8,308 pairs for further screening in human and mouse genomes, respectively.

### Screening tandem duplicates and retrogenes

Although tandem duplicates should be adjacent to each other on one chromosome, the extensive gene inversions may insert irrelevant genes into the tandem arrays. We followed the stringent definition adopted by previous studies [14,15] to screen the tandem duplicates, which restricted the inserted spacers to no more than one gene. This resulted in 1,210 and 1,802 paralogous pairs in human and mouse genomes, respectively [see additional file 1 and 2]. We implemented a method similar to those of Emerson [16] and Pan [15] to screen retrogenes. First, the pairs with a multi-exon member and an intronless member were considered as putative parental-retrogene pairs, but the pairs with both members intronless were ignored as they were not clearly created via retropositions. Next, for the putative pairs with both members located on the same chromosome, we discarded those with the intervening spacers containing less than 10 genes, since they were confused with tandem duplicates. Finally, we preserved 410 and 680 pairs resulting from retropositions in the two genomes, respectively [see additional file 3 and 4].

### Sequence similarity analysis

The similarity of protein sequences between two duplicates, as measured by their average amino acid identity, can be retrieved directly from BioMart. The *dN *and *dS *of their coding sequences were downloaded from the EPGD database http://epgd.biosino.org/EPGD/[17] [see additional file 1, 2, 3, and 4]. To avoid the influence of saturation effect [18], only the pairs with *dS *< 1 were considered in the *dN*/*dS *analysis.

### Expression similarity analysis

The tissue-specific expression profiles and the annotation of the probesets were downloaded from the GNF gene expression database http://wombat.gnf.org[19]. We chose the datasets HUMAN U133A/GNF1H and MOUSE GNF1M for the corresponding species. The Present/Absent calls in the profiles were used to indicate whether a probeset was expressed or not, and the Marginal calls were also treated as Present calls. When a gene had many probesets, it was considered to be expressed if any one of the probeset was present. We ignored the probesets such as '_f_at', '_s_at' and '_x_at' because they could not be mapped to unique genes in a gene family. For a duplicated pair, common probesets shared by the two members were also excluded. Finally, if *s *was the number of tissues where both members were expressed, and *d *was the number of tissues where one member was expressed while the other was not, then their expression similarity was calculated as *s*/(*s+d*) [see additional file 1, 2, 3, and 4].

## Results

### Gene duplicability

**Figure 1:**
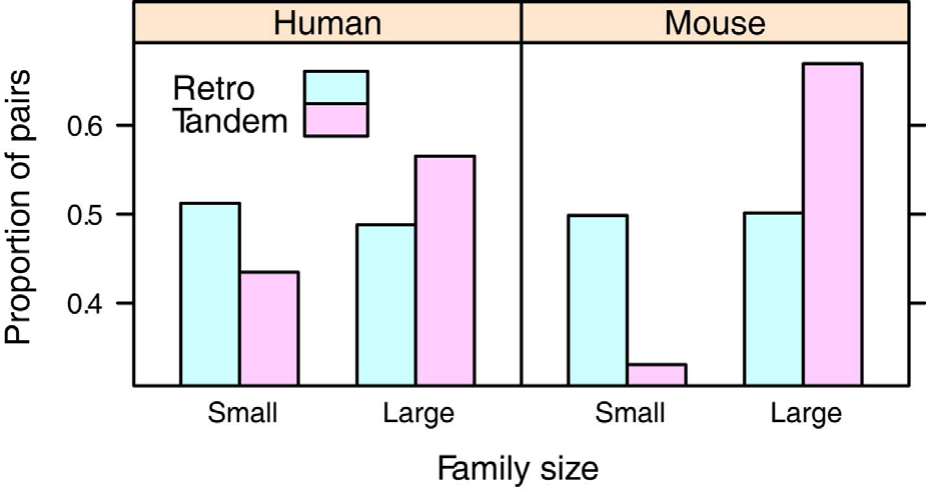
**Gene duplicability**. Distribution of the duplicates among small (≤5 members) and large (>5 members) families; tandem duplicates are more likely to be enriched in large families than the retrogenes (*p *< 0.01 for both genomes, chi-square test)

We identified 1,210 tandem duplicates and 410 retrogenes in the human genome, and 1,802 tandem duplicates and 680 retrogenes in the mouse genome. The higher number of tandem duplicates than retrogenes in both genomes implies a higher gene duplicability for tandem duplicates. Previous studies have found that gene duplicability is positively correlated with gene dosage [20] and gene complexity [21], although the correlation with functional essentiality is not always the same in yeasts and mammals [22-25]. To investigate the difference in gene duplicability between tandem duplicates and retrogenes in more detail, we counted the number of each type of duplicates in gene families with various sizes (Figure 1). The result shows that their distributions among gene families are quite different (*p *< 0.01 for both genomes, chi-square test). Specifically, tandem duplicates are more likely to be enriched in larger families, whereas retrogenes do not display a preference.

### Sequence similarity

**Figure 2:**
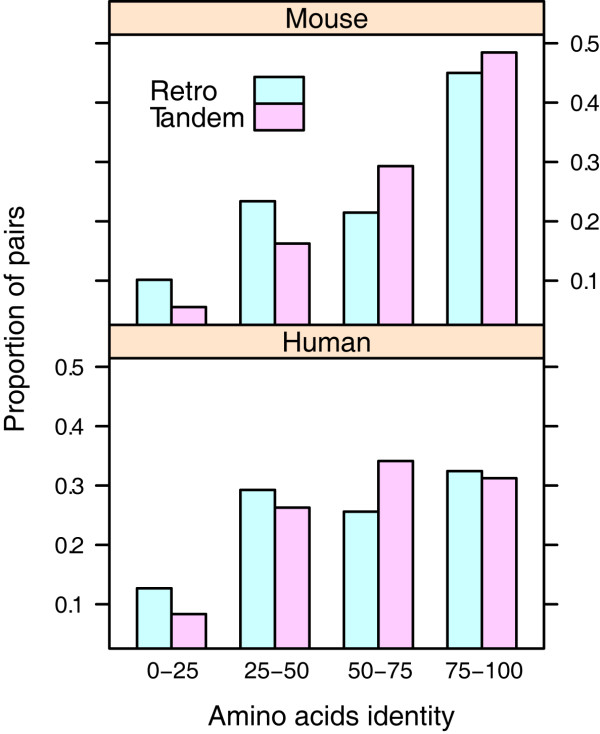
**Percentage of amino acid identity**. Medians for tandem duplicates and retrogenes in mouse genomes are 74 and 68.75, respectively (*p *= 0.034, one-tailed rank sum test); medians for both types of duplicates in human genomes are 63 and 55.75, respectively (*p *= 0.021)

Similarity of coding sequences has been widely used to indicate whether the new duplicates undergo gene conservation or functional divergence. While some reports have suggested that the duplicates really undergo sequence divergence when they are newly produced [9,26], other reports have found that they still remain more conserved than singletons [27]. However, taking all duplicates as a whole will neglect some specific factors that belong to different molecular bases. For example, the effect of gene conversion, which keeps duplicates appearing similar through local DNA recombination [28], may have greater influence on tandem duplicates than retrogenes. The higher duplicability of tandem duplicates may also leave more recent and less divergent gene pairs. To test the hypothesis, we compared the amino acid identity between both types of duplicates (Figure 2). The result shows that, the sequence identity of tandem duplicates is significantly higher than that of retrogenes (human: *p *= 0.021, mouse: *p *= 0.034, rank sum test). In agreement with Jun et al. [7], this result implies that tandem duplicates tend to be more conserved, whereas retrogenes tend to be more divergent.

### Expression similarity

**Figure 3:**
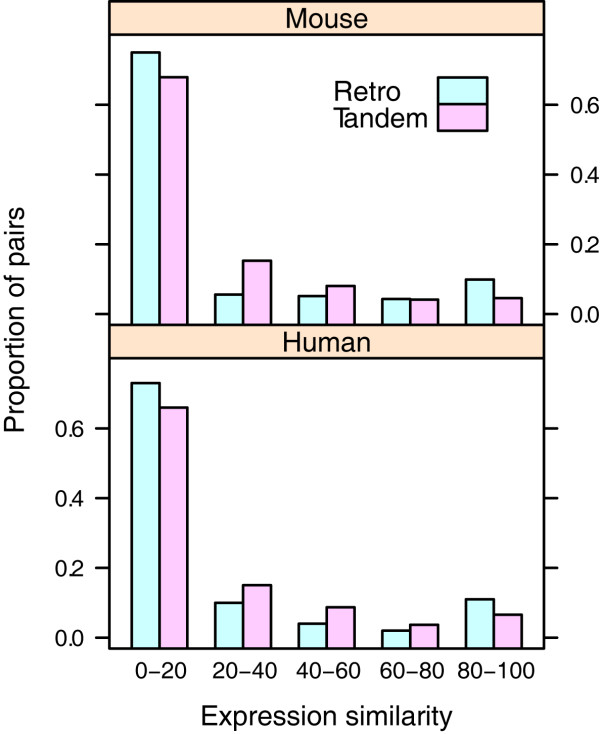
**Percentage of tissues with duplicates co-expressed**. Medians for tandem duplicates and retrogenes in mouse genomes are 8.5 and 1.8, respectively (*p *= 0.002, one-tailed rank sum test); medians for the both of duplicates in human genomes are 7.9 and 2.0, respectively (*p *= 0.036)

In addition to the coding sequences, the evolution of regulatory elements is also important to determine the fate of duplicates. In fact, the differentiation of regulatory motifs can increase the expression specificity of the duplicates among various tissues and developmental stages, which is perhaps the most common form of subfunctionalization [29]. Previous reports have found that a rapid expression divergence exists between duplicates [30], and that the expression diversity is also increased compared to singletons [31]. However, as tandem duplications directly occur at the DNA level, it is more likely that the new duplicates preserve their original regulatory motifs and expression patterns. In contrast, as retrogenes are randomly inserted into the genome via mRNAs, they are more likely to acquire distinct regulatory motifs and expression patterns. To test this hypothesis, we compared the expression similarity for tandem duplicates and retrogenes by using microarray data across diverse tissues (Figure 3). Although a lot of duplicates have been quite differentiated for both types, the expression similarity between tandem duplicates is still significantly higher than that between retrogenes (human: *p *= 0.036, mouse: *p *= 0.002, rank sum test). Therefore, the gene expression profiles also support the difference in functional divergence for both types of duplications.

### Role of positive selection

**Figure 4:**
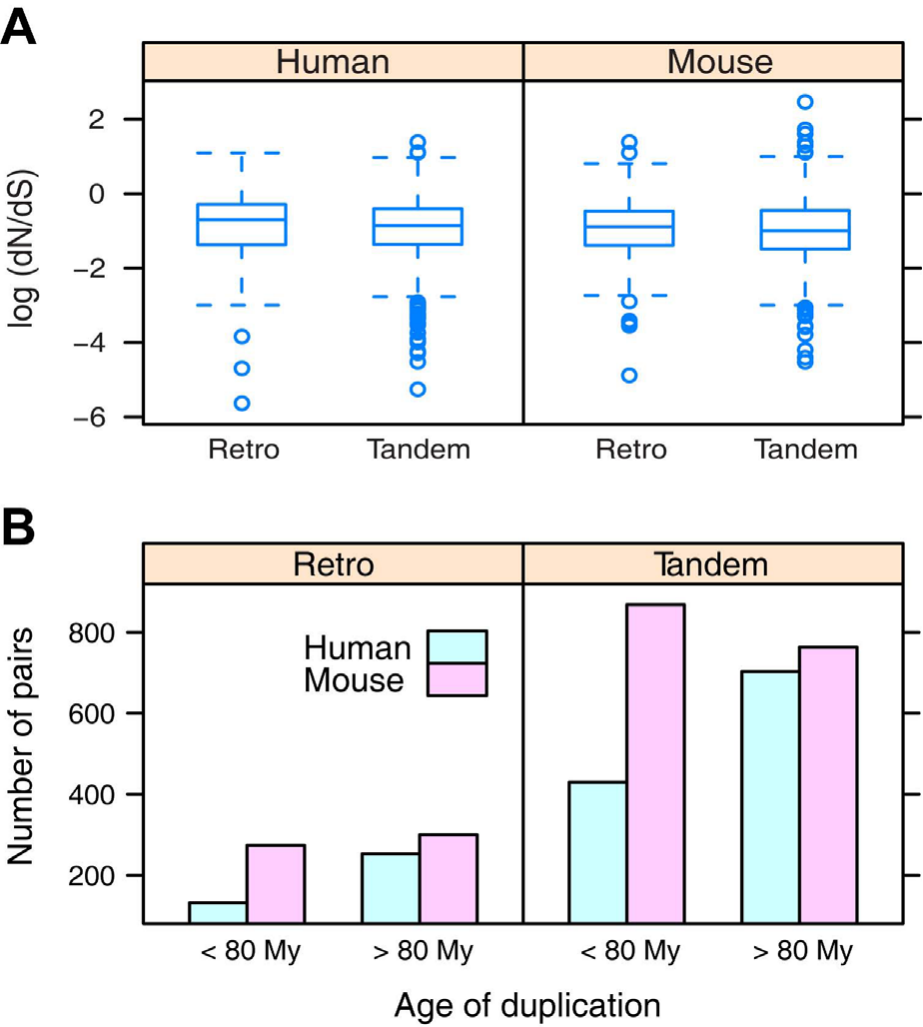
**Role of positive selection**. (A) Comparison of *dN/dS *ratios for tandem duplicates and retrogenes (log-scale). The non-synonymous substitutions in most duplicates are under selective constraints (*dN/dS *< 1); there is little difference in the *dN/dS *ratios between both types of duplicates (human: *p *= 0.607, mouse: *p*= 0.257, *t*-test) (B) Distribution of the duplicates before and after the split of human and mouse lineages (≈80 million years ago [33]). *dS*/2 was used to estimate the duplication age, which can be translated to the absolute time scale by using 2.5e-3 substitutions per site per million years [9]; there are significantly more tandem duplicates and retrogenes arising in the mouse-specific lineage (*p *< 1e-4 for both types, chi-square test)

As mentioned in the section **Background**, the retention mechanisms are determined by both functional divergence and evolutionary forces. To compare the evolutionary forces for both types of duplicates, we first performed the traditional *dN*/*dS *analysis (Figure 4A). The result shows little difference in the *dN*/*dS *ratios between tandem duplicates and retrogenes (human: *p *= 0.607, mouse: *p *= 0.257, *t*-test). In addition, the non-synonymous substitutions in most duplicates are under selective constraints (*dN*/*dS *< 1).

Nonetheless, the *dN*/*dS *test is directed to single site substitutions, which is not suitable for the case of whole gene substitutions such as the addition of duplicates. Lynch [32] has presented a new strategy for this issue by examining the role of effective population size. Briefly, if the new duplicates are nearly neutral and fixed by genetic drift, a small population size is favourable for their retention. On the contrary, if the new duplicates are advantageous and fixed by positive selection, the opposite should be true. In fact, Lynch has suggested that the long-term increase of duplicates from prokaryotes to eukaryotes is initially a neutral process in response to the reduction of population size [32]. However, Shiu et al. [33] have argued that positive selection also plays an important role at least in mammalian genomes because there are more duplicates retained in the mouse lineage (larger population size) than in the human lineage (smaller population size), which cannot be explained by the difference in their duplication rate. Furthermore, since the generation time in mice is shorter than in humans, there will be more generations that are subject to selective pressures for mice and consequently, more duplicates retained in the mouse genome. In our dataset, there are both more tandem duplicates and more retrogenes in the mouse genome. To test if the excessive duplicates are really created in the mouse lineage, we grouped the age of the duplicates (inferred from *dS*) according to the divergence time between the two species (Figure 4B). The result shows that, while the duplicates generated prior to the split of the two genomes are more or less the same, there are more duplicates arising in the mouse-specific lineage (*p *< 1e-4 for both types, chi-square test). Based on the same assumption with Shiu et al. [33], this result implies that positive selection plays essential roles in the retention of both types of duplicates.

## Discussion

### Dosage effect is more prevalent in tandem duplicates

Of the two key dimensions to determine the retention mechanisms, we have found that the extent of functional divergence is distinct for tandem duplicates and retrogenes, whereas the underlying evolutionary forces are the same. As tandem duplicates are generated at the DNA level and easily influenced by gene conversion, they are more likely to be maintained (Figure 2 and 3). Two main models can be used to account for the conservation of duplicates, i.e. dosage model and buffering model. The former proposes that as the new duplicates will increase the gene dosage, they can bring about some selective advantages [20]. In contrast, the latter argues that the conserved duplicates are just used for compensation in case of the functional loss of their counterparts [34], and thus they are free from selective pressures. Given the signature of positive selection (Figure 4), we propose that the dosage model is more prevalent in the fixation of tandem duplicates. In fact, the dosage model predicts that the fitness of dosage-sensitive genes will increase with the increase of gene copies [20], which is consistent with our observation that tandem duplicates tend to form large families (Figure 1). Another large-scale functional analysis has revealed that tandem duplicates are enriched in receptors and binding proteins [14], which are also dosage-sensitive genes [20]. Interestingly, copy number variants (CNV), which are strongly associated with segmental tandem duplicates [35], may also be maintained by dosage effect and positive selection [36].

### EAC effect is more prevalent in retrogenes

Retrogenes and tandem duplicates display nearly opposite molecular properties. Since retrogenes are often distant from their parental counterparts and lose the original regulatory elements, they are more likely to undergo functional divergence (Figure 2 and 3). There are also two main models available to account for the functional divergence, namely 'escape from adaptive conflict' (EAC) model [37] and 'duplication-degeneration-complementation' (DDC) model [38]. Both of the models predict that the new duplicates will share the functions of the ancestral genes. However, the EAC model argues that duplications can release the potential benefits through functional specialization, whereas the DDC model only requires that the joint effect of the duplicates fulfil the original functions. The signature of positive selection in the retention of retrogenes votes for the prevalence of the EAC model (Figure 4). In addition to our results, the analysis of gene movements has revealed that the X-linked genes are excessively transferred to autosomes via retropositions in mammalian genomes [16]. These retrogenes can not only sustain essential functions during the inactivation of the male X chromosome, but also develop male-specific expression patterns [16,39]. The coexistence of functional divergence and selective benefits provides an important evidence for the EAC model.

## Competing interests

The authors declare that they have no competing interests.

## Authors' contributions

ZW conceived and performed the experiments. XD participated in the discussions. ZW and GD collected the data. ZW, GD and YL wrote and revised the manuscript.

All authors read and approved the final manuscript.

## Supplementary Material

Additional file 1 **Human tandem duplicates**. Click here for file

Additional file 2 **Mouse tandem duplicates**. Click here for file

Additional file 3 **Human retrogenes**. Click here for file

Additional file 4 **Mouse retrogenes**. Click here for file

## References

[B1] Long M Betran E Thornton K Wang W The origin of new genes: glimpses from the young and old Nat Rev Genet 2003 4 865 875 10.1038/nrg1204 14634634

[B2] Zhang JZ Evolution by gene duplication: an update Trends Ecol Evol 2003 18 292 298 10.1016/S0169-5347(03)00033-8

[B3] Hahn MW Distinguishing among evolutionary models for the maintenance of gene duplicates J Hered 2009 100 605 617 10.1093/jhered/esp047 19596713

[B4] Durand D Hoberman R Diagnosing duplications--can it be done? Trends Genet 2006 22 156 164 10.1016/j.tig.2006.01.002 16442663

[B5] Conrad B Antonarakis SE Gene duplication: a drive for phenotypic diversity and cause of human disease Annu Rev Genomics Hum Genet 2007 8 17 35 10.1146/annurev.genom.8.021307.110233 17386002

[B6] Lynch M Katju V The altered evolutionary trajectories of gene duplicates Trends Genet 2004 20 544 549 10.1016/j.tig.2004.09.001 15475113

[B7] Jun J Ryvkin P Hemphill E Nelson C Duplication mechanism and disruptions in flanking regions determine the fate of Mammalian gene duplicates J Comput Biol 2009 16 1253 1266 10.1089/cmb.2009.0074 19772436

[B8] Wang Z Ding G Yu Z Liu L Li Y Modeling the age distribution of gene duplications in vertebrate genome using mixture density Genomics 2009 93 146 151 10.1016/j.ygeno.2008.10.008 19027062

[B9] Lynch M Conery JS The evolutionary fate and consequences of duplicate genes Science 2000 290 1151 1155 10.1126/science.290.5494.1151 11073452

[B10] Lynch M Conery JS The evolutionary demography of duplicate genes J Struct Funct Genomics 2003 3 35 44 10.1023/A:1022696612931 12836683

[B11] Flicek P Aken BL Beal K Ballester B Caccamo M Chen Y Clarke L Coates G Cunningham F Cutts T Down T Dyer SC Eyre T Fitzgerald S Fernandez-Banet J Graf S Haider S Hammond M Holland R Howe KL Howe K Johnson N Jenkinson A Kahari A Keefe D Kokocinski F Kulesha E Lawson D Longden I Megy K Ensembl 2008 Nucleic Acids Res 2008 36 Database D707 714 18000006 10.1093/nar/gkm988 PMC2238821

[B12] Wang Z Ding G Yu Z Liu L Li Y CHSMiner: a GUI tool to identify chromosomal homologous segments Algorithms Mol Biol 2009 4 2 10.1186/1748-7188-4-2 19146671 PMC2647922

[B13] McLysaght A Hokamp K Wolfe KH Extensive genomic duplication during early chordate evolution Nat Genet 2002 31 200 204 10.1038/ng884 12032567

[B14] Shoja V Zhang L A roadmap of tandemly arrayed genes in the genomes of human, mouse, and rat Mol Biol Evol 2006 23 2134 2141 10.1093/molbev/msl085 16901985

[B15] Pan D Zhang L Quantifying the major mechanisms of recent gene duplications in the human and mouse genomes: a novel strategy to estimate gene duplication rates Genome Biol 2007 8 R158 10.1186/gb-2007-8-8-r158 17683522 PMC2374989

[B16] Emerson JJ Kaessmann H Betran E Long M Extensive gene traffic on the mammalian X chromosome Science 2004 303 537 540 10.1126/science.1090042 14739461

[B17] Ding G Sun Y Li H Wang Z Fan H Wang C Yang D Li Y EPGD: a comprehensive web resource for integrating and displaying eukaryotic paralog/paralogon information Nucleic Acids Res 2008 36 Database D255 262 17984073 10.1093/nar/gkm924 PMC2238967

[B18] Smith JM Smith NH Synonymous nucleotide divergence: what is "saturation"? Genetics 1996 142 1033 1036 8849908 10.1093/genetics/142.3.1033 PMC1207002

[B19] Su AI Wiltshire T Batalov S Lapp H Ching KA Block D Zhang J Soden R Hayakawa M Kreiman G Cooke MP Walker JR Hogenesch JB A gene atlas of the mouse and human protein-encoding transcriptomes Proc Natl Acad Sci USA 2004 101 6062 6067 10.1073/pnas.0400782101 15075390 PMC395923

[B20] Kondrashov FA Koonin EV A common framework for understanding the origin of genetic dominance and evolutionary fates of gene duplications Trends Genet 2004 20 287 290 10.1016/j.tig.2004.05.001 15219392

[B21] He X Zhang J Gene complexity and gene duplicability Curr Biol 2005 15 1016 1021 10.1016/j.cub.2005.04.035 15936271

[B22] He X Zhang J Higher duplicability of less important genes in yeast genomes Mol Biol Evol 2006 23 144 151 10.1093/molbev/msj015 16151181

[B23] Prachumwat A Li WH Protein function, connectivity, and duplicability in yeast Mol Biol Evol 2006 23 30 39 10.1093/molbev/msi249 16120800

[B24] Liang H Li WH Gene essentiality, gene duplicability and protein connectivity in human and mouse Trends Genet 2007 23 375 378 10.1016/j.tig.2007.04.005 17512629

[B25] Liao BY Zhang J Mouse duplicate genes are as essential as singletons Trends Genet 2007 23 378 381 10.1016/j.tig.2007.05.006 17559966

[B26] Zhang P Gu Z Li WH Different evolutionary patterns between young duplicate genes in the human genome Genome Biol 2003 4 R56 10.1186/gb-2003-4-9-r56 12952535 PMC193656

[B27] Davis JC Petrov DA Preferential duplication of conserved proteins in eukaryotic genomes PLoS Biol 2004 2 E55 10.1371/journal.pbio.0020055 15024414 PMC368158

[B28] Gao LZ Innan H Very low gene duplication rate in the yeast genome Science 2004 306 1367 1370 10.1126/science.1102033 15550669

[B29] Li WH Yang J Gu X Expression divergence between duplicate genes Trends Genet 2005 21 602 607 10.1016/j.tig.2005.08.006 16140417

[B30] Gu Z Nicolae D Lu HH Li WH Rapid divergence in expression between duplicate genes inferred from microarray data Trends Genet 2002 18 609 613 10.1016/S0168-9525(02)02837-8 12446139

[B31] Gu Z Rifkin SA White KP Li WH Duplicate genes increase gene expression diversity within and between species Nat Genet 2004 36 577 579 10.1038/ng1355 15122255

[B32] Lynch M Conery JS The origins of genome complexity Science 2003 302 1401 1404 10.1126/science.1089370 14631042

[B33] Shiu SH Byrnes JK Pan R Zhang P Li WH Role of positive selection in the retention of duplicate genes in mammalian genomes Proc Natl Acad Sci USA 2006 103 2232 2236 10.1073/pnas.0510388103 16461903 PMC1413713

[B34] Gu Z Steinmetz LM Gu X Scharfe C Davis RW Li WH Role of duplicate genes in genetic robustness against null mutations Nature 2003 421 63 66 10.1038/nature01198 12511954

[B35] Redon R Ishikawa S Fitch KR Feuk L Perry GH Andrews TD Fiegler H Shapero MH Carson AR Chen W Cho EK Dallaire S Freeman JL Gonzalez JR Gratacos M Huang J Kalaitzopoulos D Komura D MacDonald JR Marshall CR Mei R Montgomery L Nishimura K Okamura K Shen F Somerville MJ Tchinda J Valsesia A Woodwark C Yang F Global variation in copy number in the human genome Nature 2006 444 444 454 10.1038/nature05329 17122850 PMC2669898

[B36] Nguyen DQ Webber C Ponting CP Bias of selection on human copy-number variants PLoS Genet 2006 2 e20 10.1371/journal.pgen.0020020 16482228 PMC1366494

[B37] Storz JF Genome evolution: gene duplication and the resolution of adaptive conflict Heredity 2009 102 99 100 10.1038/hdy.2008.114 18971957 PMC4390061

[B38] Force A Lynch M Pickett FB Amores A Yan YL Postlethwait J Preservation of duplicate genes by complementary, degenerative mutations Genetics 1999 151 1531 1545 10101175 10.1093/genetics/151.4.1531 PMC1460548

[B39] Vinckenbosch N Dupanloup I Kaessmann H Evolutionary fate of retroposed gene copies in the human genome Proc Natl Acad Sci USA 2006 103 3220 3225 10.1073/pnas.0511307103 16492757 PMC1413932

